# Supplementary material for: Pathophysiological Significance of Neutrophilic Transfer RNA-Derived Small RNAs in Asymptomatic Moyamoya Disease
Source: Cells. 2021 May 1;10(5):1086. doi: 10.3390/cells10051086 (PMC8147334; doi:10.3390/cells10051086)
Supplement: Supplementary file 1 [file cells-10-01086-s001.zip › cells-1149161 3rd supplementary materials/Data Supplements/Data Supplement V.zip!/Data supplement 6-mRNAs targets of tsRNAs/tsRNA-Val-AAC-017/Diagrams/tRF-Val-AAC-017_vs_ENCT00000424376(CATG00000098647.1).pdf]

2D Structure	Local AU	Position
	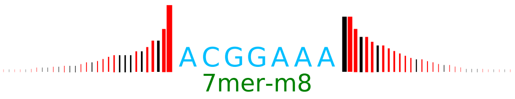	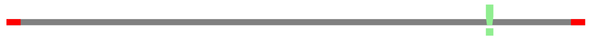

```
822                          851
                          7mer-m8
5'-acACCTAGATAACCGACTAGA-TACGGAAAg-3' utr
      || |  ||||||| ||||  | |||||||
3'-uuUGCA-CUAUUGG-UGAUGUGAUGCCUUUg-5' tRF
                  16151413   7 6 5 4 3 2
               3' Pairing      Seed
```
